# Supplementary material for: Highly Oxygenated Constituents from a Marine Alga-Derived Fungus Aspergillus giganteus NTU967
Source: Mar Drugs. 2020 Jun 6;18(6):303. doi: 10.3390/md18060303 (PMC7374281; doi:10.3390/md18060303)
Supplement: Supplementary file 1 [file marinedrugs-18-00303-s001.pdf]

Supporting Information
for
Highly Oxygenated Constituents from A Marine Alga-derived Fungus *Aspergillus giganteus* NTU967

Jih-Jung Chen, Shih-Wei Wang, Yin-Ru Chiang, Ka-Lai Pang, Yueh-Hsiung Kuo, Tsai-Yen Shih, Tzong-Huei Lee

Page 3, Figure S1. ^{1}H NMR (400 MHz, MeOH-d_4) of **1**.
Figure S2. ^{13}C NMR (125 MHz, MeOH-d_4) of **1**.
Page 4, Figure S3. HSQC of **1**.
Figure S4. COSY of **1**.
Page 5, Figure S5. HMBC of **1**.
Page 6, Figure S6. ^{1}H NMR (500 MHz, MeOH-d_4) of **2**.
Figure S7. ^{13}C NMR (125 MHz, MeOH-d_4) of **2**.
Page 7, Figure S8. HSQC of **2**.
Figure S9. COSY of **2**.
Page 8, Figure S10. HMBC of **2**.
Page 9, Figure S11. ^{1}H NMR (500 MHz, MeOH-d_4) of **3**.
Figure S12. ^{13}C NMR (125 MHz, MeOH-d_4) of **3**.
Page 10, Figure S13. HSQC of **3**.
Figure S14. COSY of **3**.
Page 11, Figure S15. HMBC of **3**.
Page 12, Figure S16. ^{1}H NMR (500 MHz, MeOH-d_4) of **4**.
Figure S17. ^{13}C NMR (125 MHz, MeOH-d_4) of **4**.
Page 13, Figure S18. HSQC of **4**.
Figure S19. COSY of **4**.
Page 14, Figure S20. HMBC of **4**.
Page 15, Figure S21. ^{1}H NMR (500 MHz, MeOH-d_4) of **5**.
Figure S22. ^{13}C NMR (125 MHz, MeOH-d_4) of **5**.
Page 16, Figure S23. HSQC of **5**.
Figure S24. COSY of **5**.
Page 17, Figure S25. HMBC of **5**.
Figure S26. ROESY of **5**.
Page 18, Figure S27. ^{1}H NMR (500 MHz, MeOH-d_4) of **6**.
Figure S28. ^{13}C NMR (125 MHz, MeOH-d_4) of **6**.
Page 19, Figure S29. HSQC of **6**.
Figure S30. COSY of **6**.

Page 20, Figure S31. HMBC of **6**.
Figure S32. ROESY of **6**.
Page 21, Figure S33. ^{1}H NMR (500 MHz, MeOH-d_4) of **7**.
Figure S34. ^{13}C NMR (125 MHz, MeOH-d_4) of **7**.
Page 22, Figure S35. HSQC of **7**.
Figure S36. COSY of **7**.
Page 23, Figure S37. HMBC of **7**.
Figure S38. ROESY of **7**.
Page 24, Figure S39. The structures of known compounds isolated in this study.
Page 25, Table S1. Cytotoxicities of aspergilsmins A-G (**1**-**7**), patulin, deoxytryptoquivaline, tryptoquivaline, and quinadoline B against PC-3 and SK-Hep-1 cells.

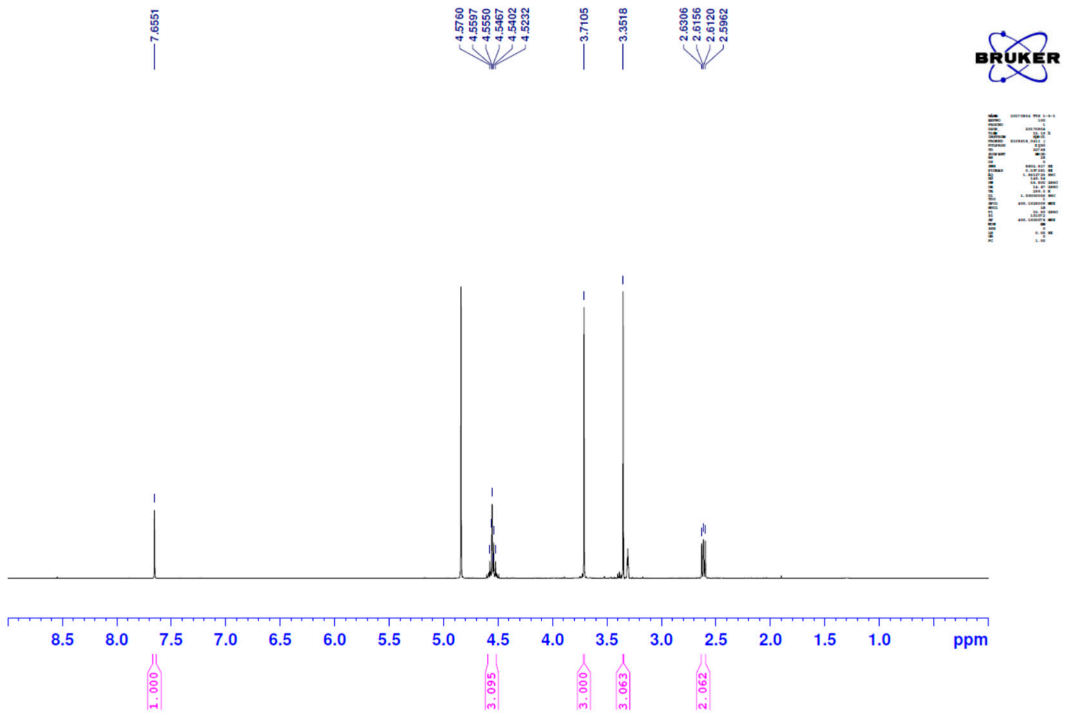

Figure S1. ^{1}H NMR (400 MHz, MeOH-d_4) of **1**.

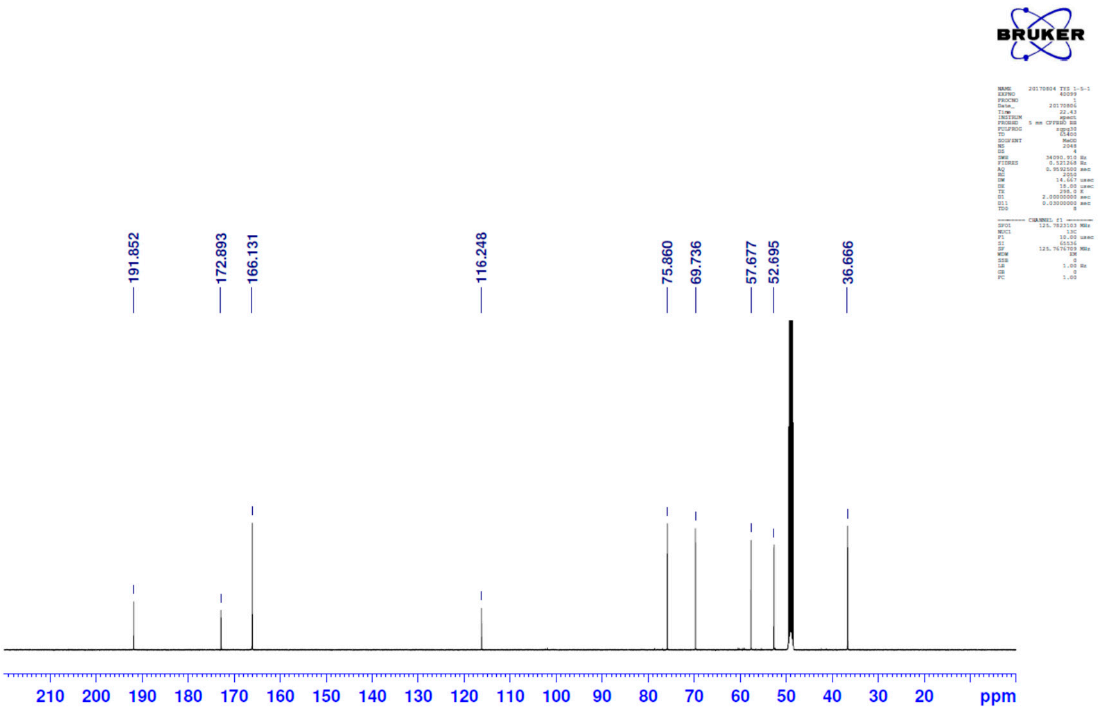

Figure S2. ^{13}C NMR (125 MHz, MeOH-d_4) of **1**.

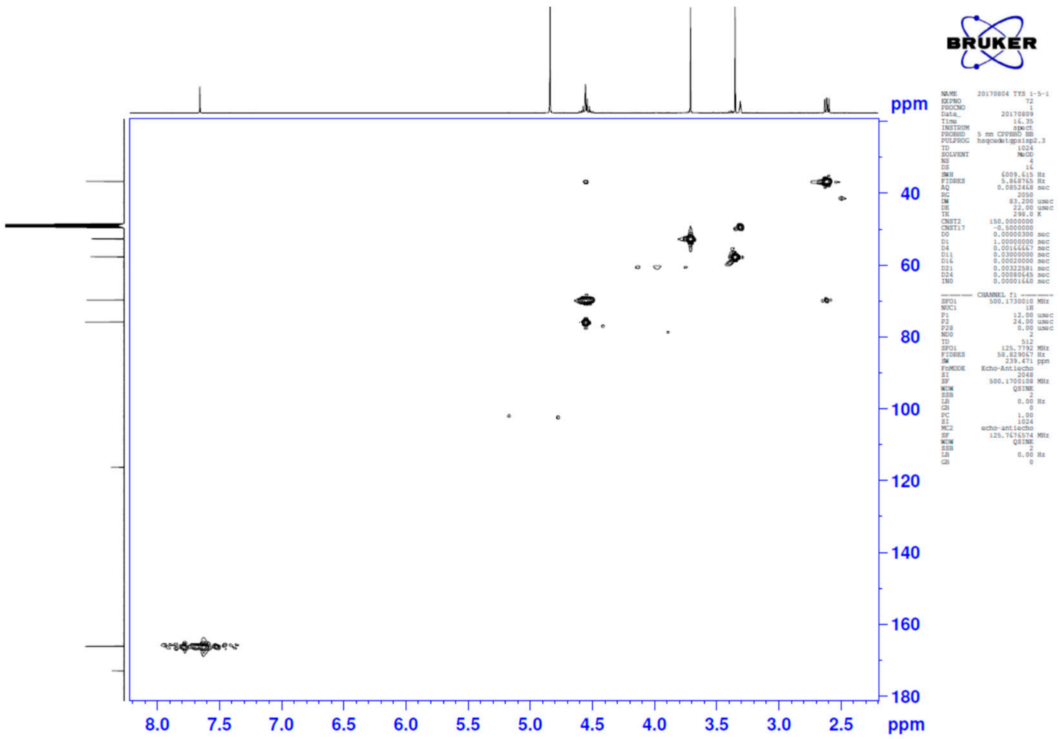

Figure S3. HSQC of **1**.

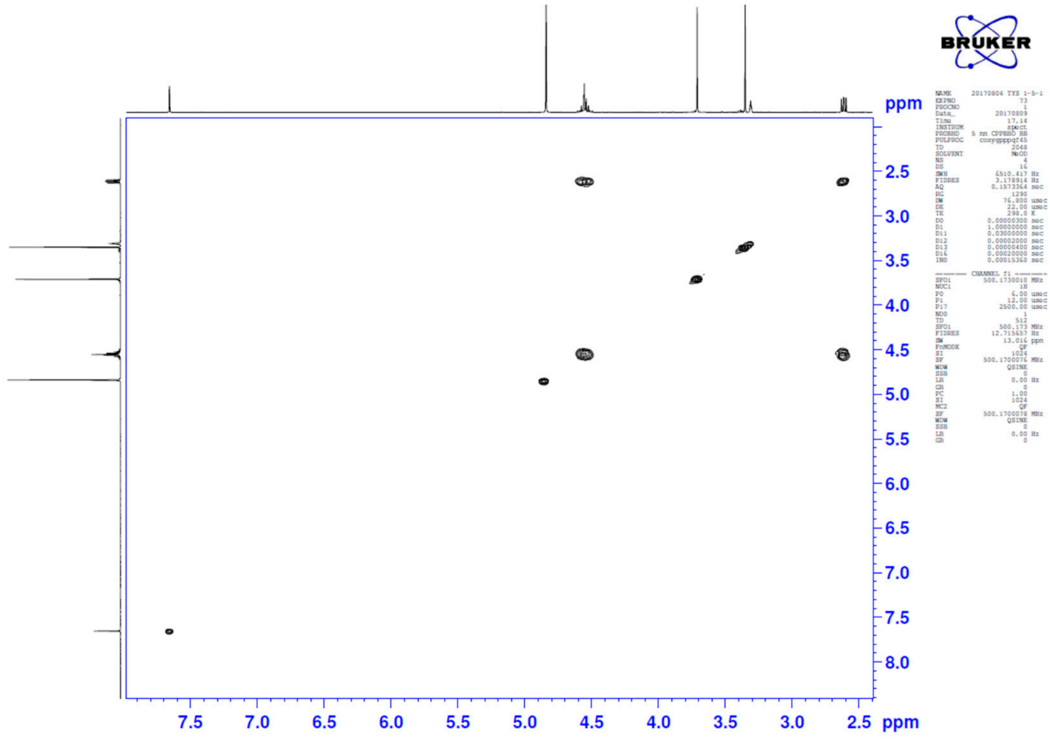

Figure S4. COSY of **1**.

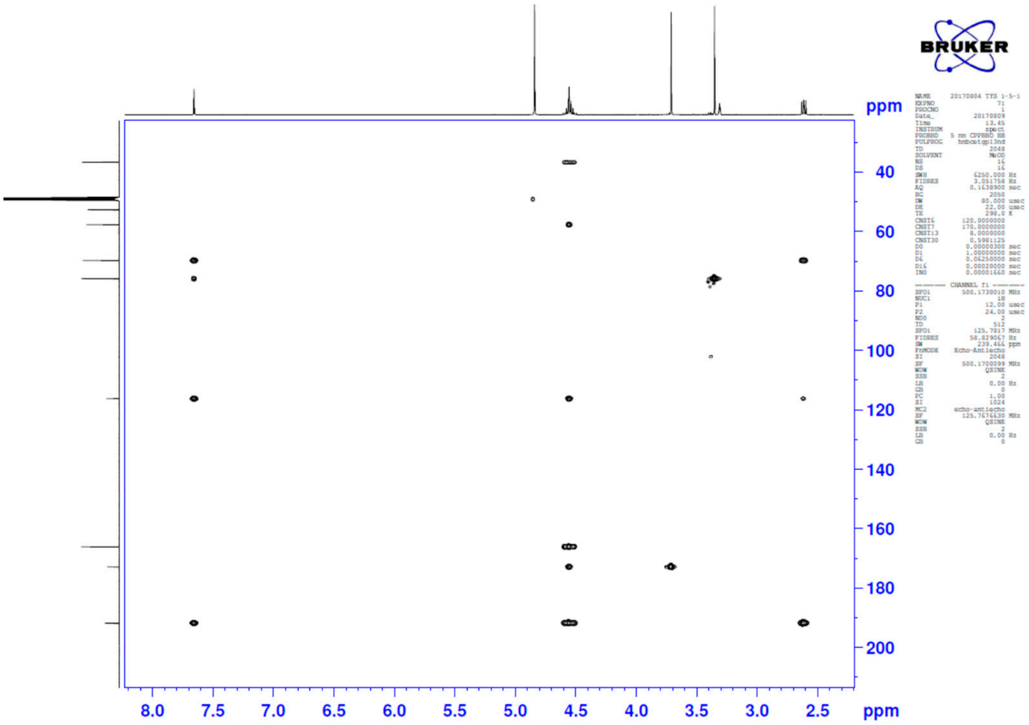

Figure S5. HMBC of **1**.

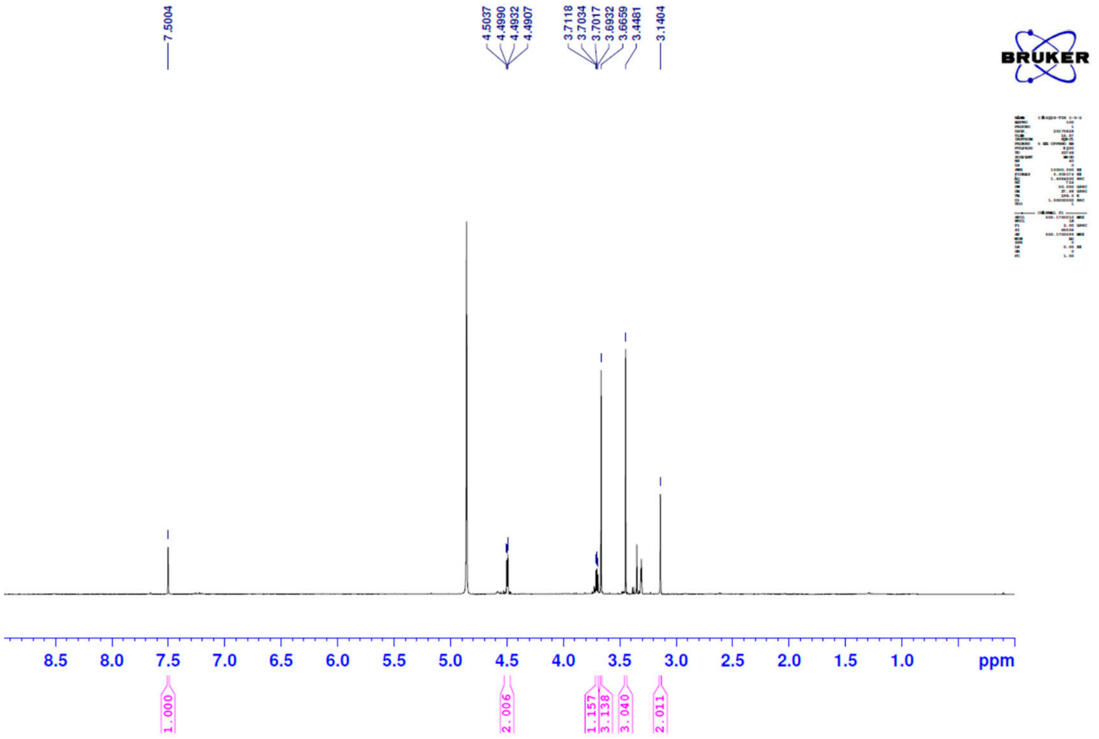

Figure S6. ^{1}H NMR (500 MHz, MeOH-d_4) of **2**.

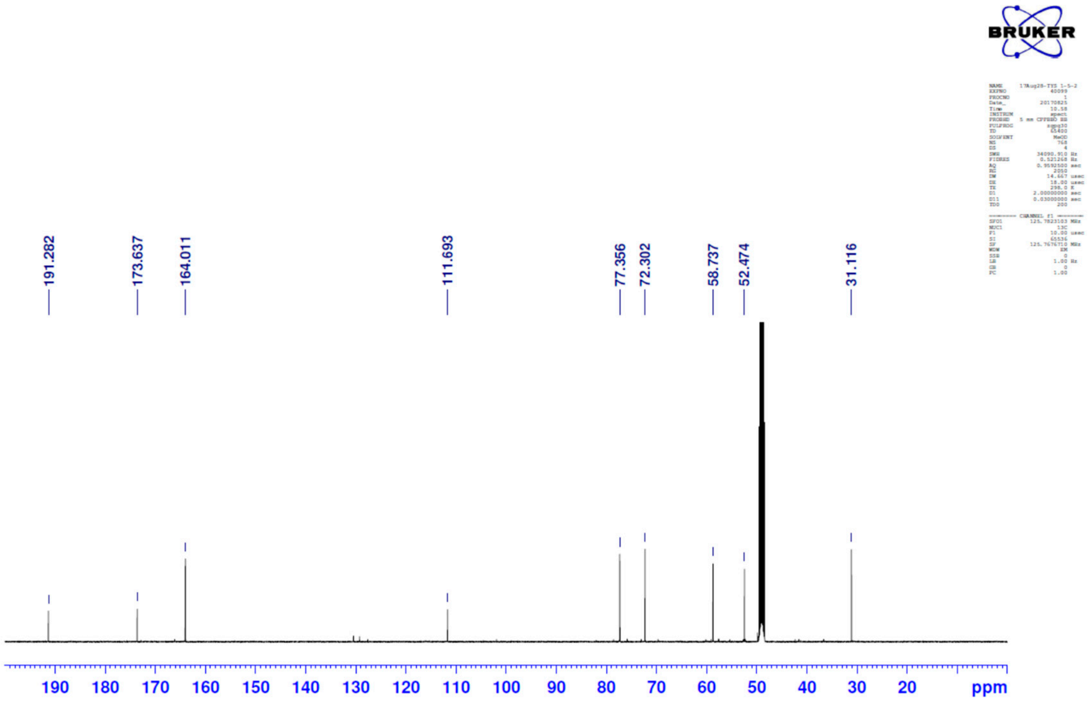

Figure S7. ^{13}C NMR (125 MHz, MeOH-d_4) of **2**.

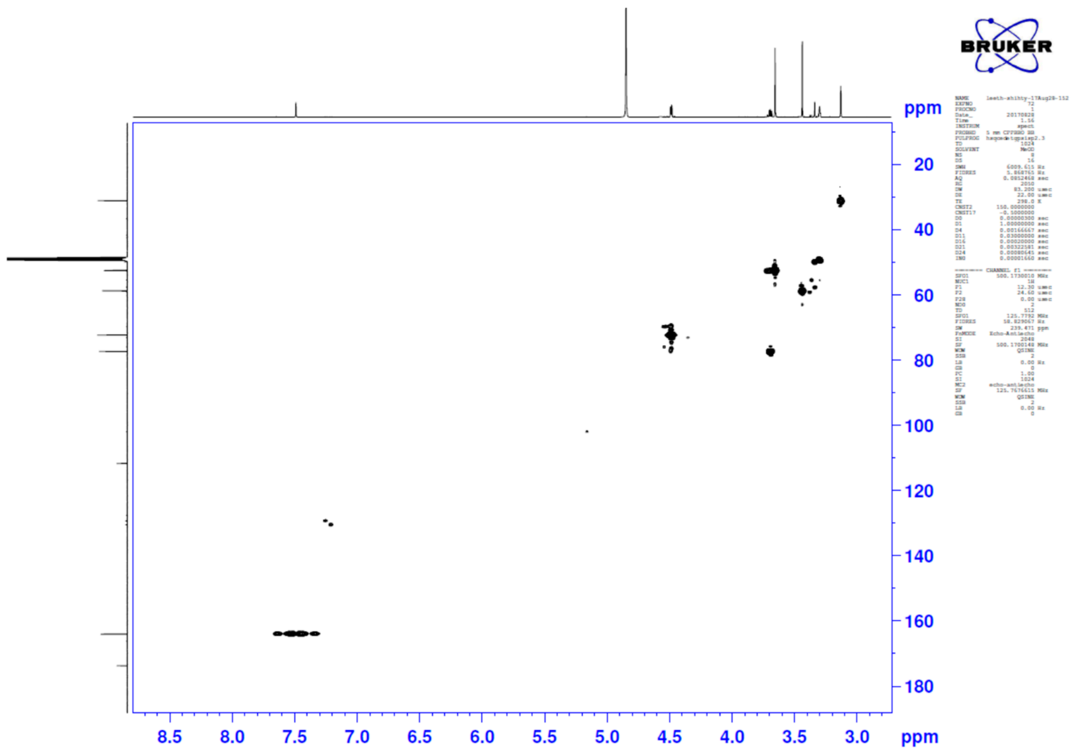

Figure S8. HSQC of **2**.

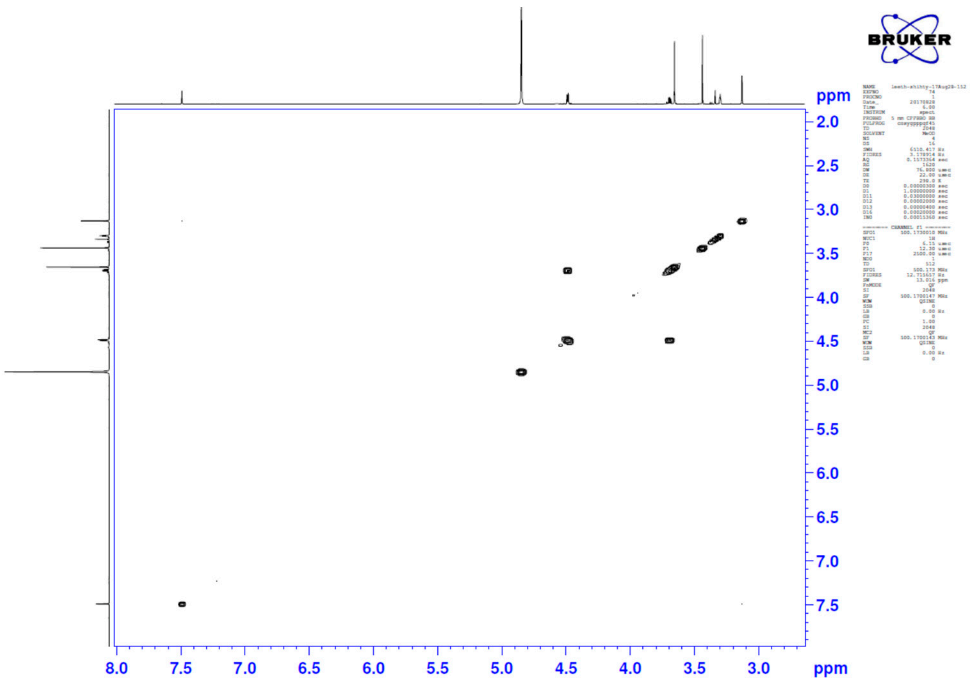

Figure S9. COSY of **2**.

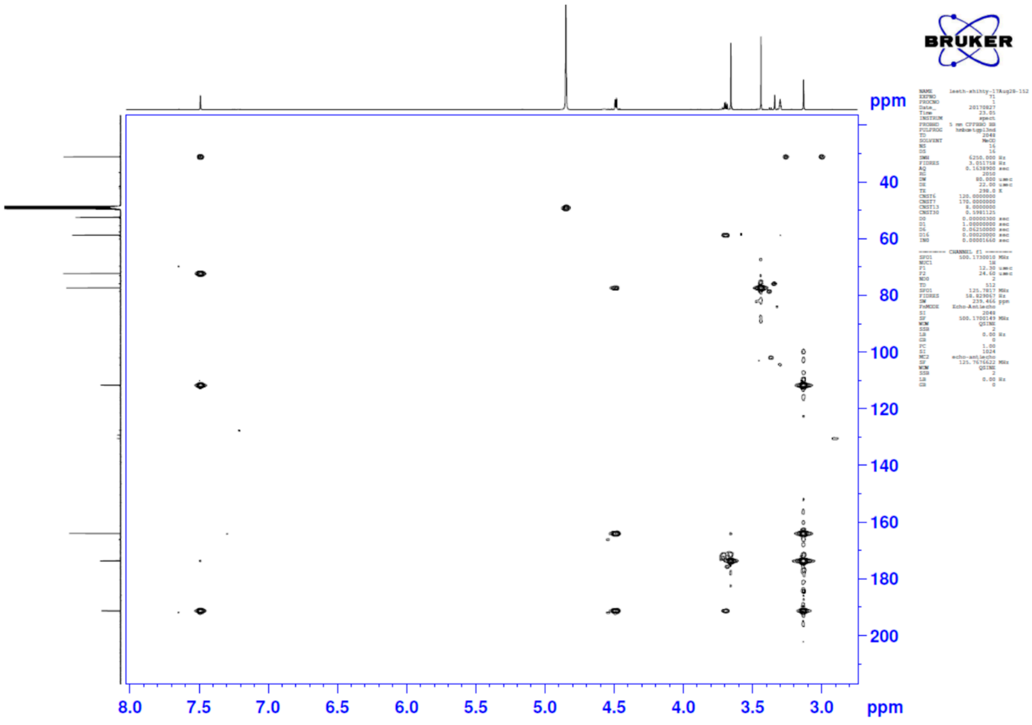

Figure S10. HMBC of **2**.

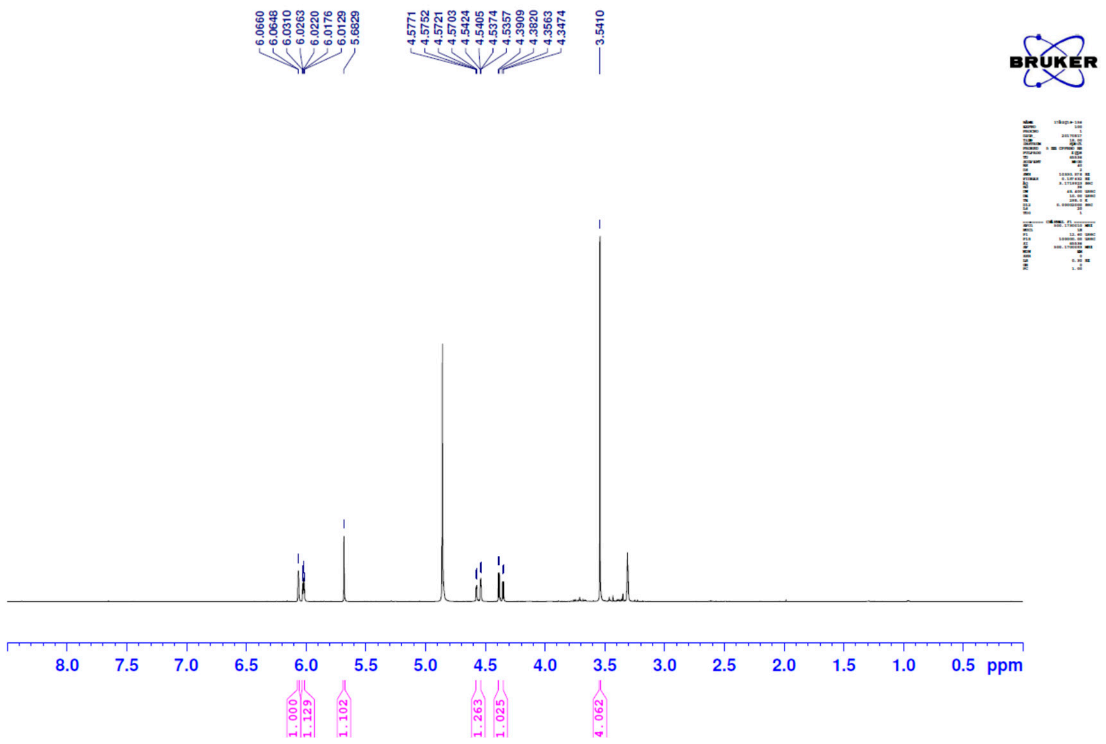

Figure S11. ^{1}H NMR (500 MHz, MeOH-d_4) of **3**.

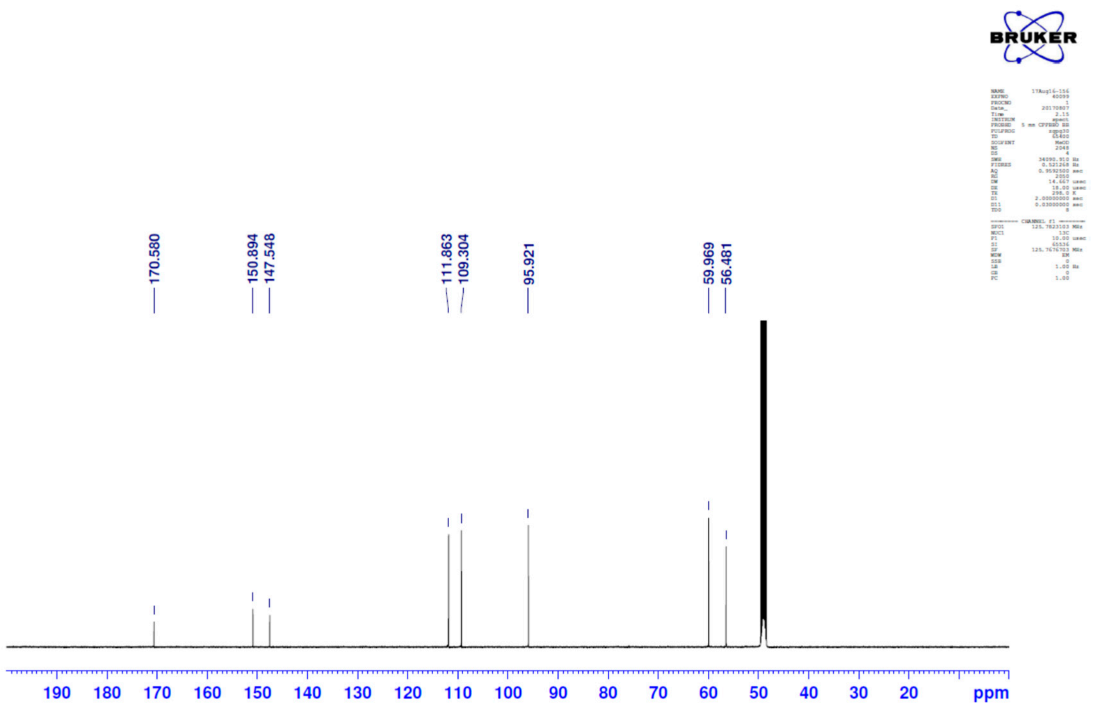

Figure S12. ^{13}C NMR (125 MHz, MeOH-d_4) of **3**.

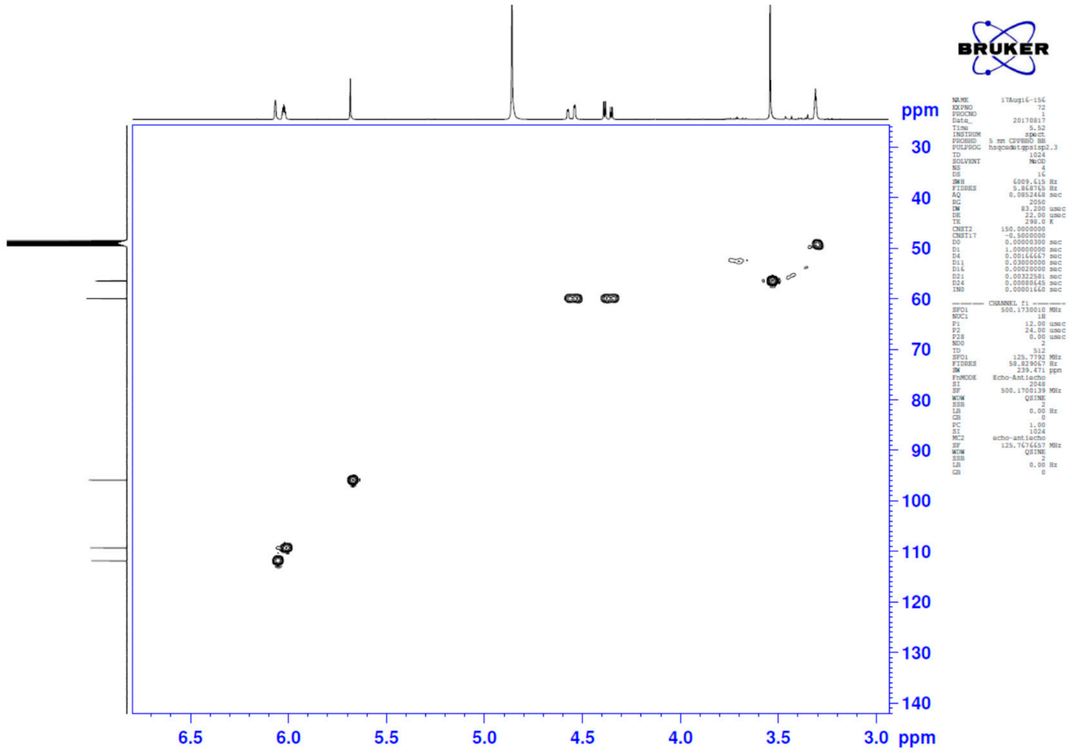

Figure S13. HSQC of **3**.

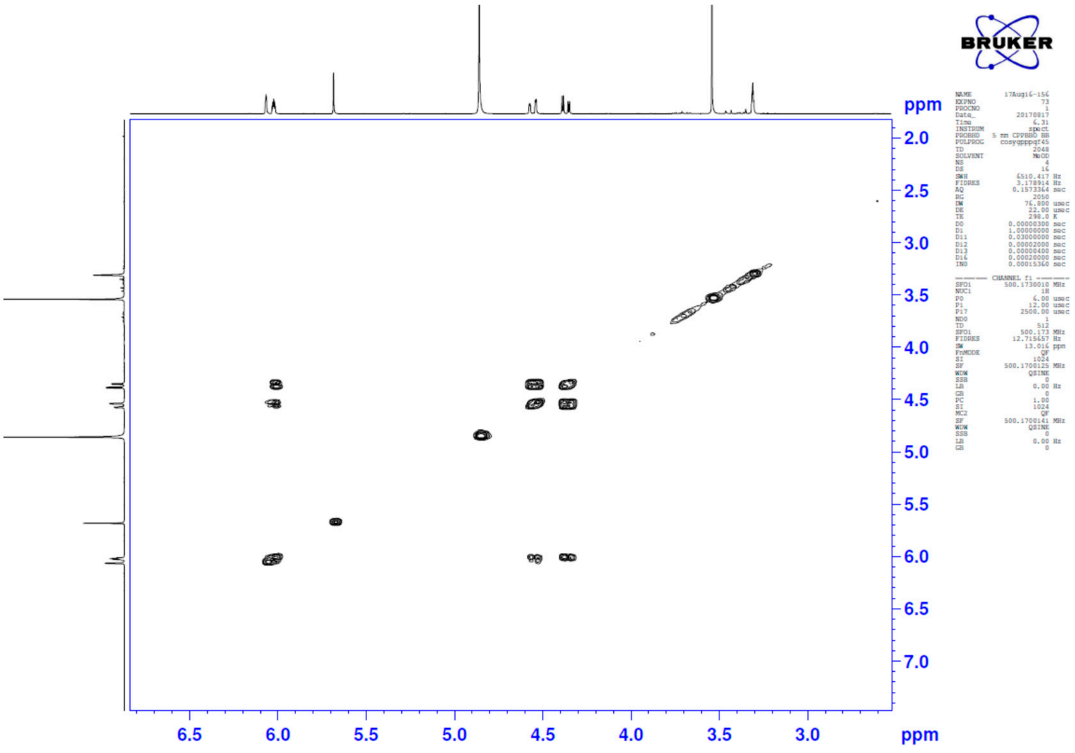

Figure S14. COSY of **3**.

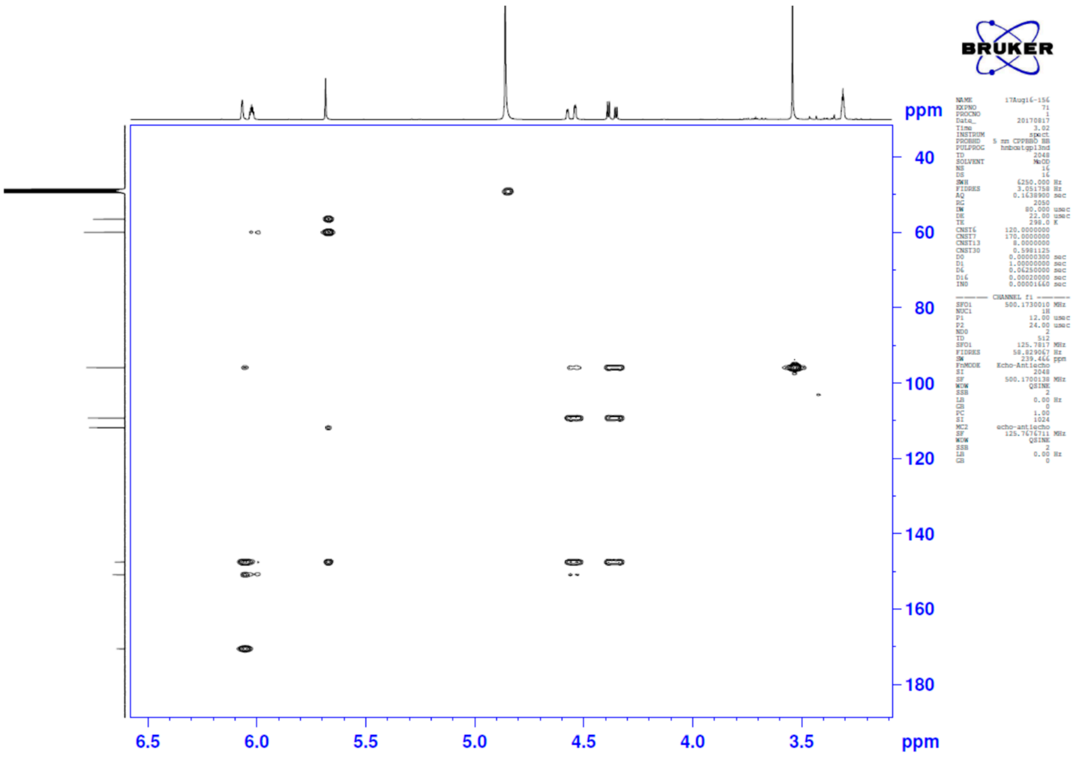

Figure S15. HMBC of **3**.

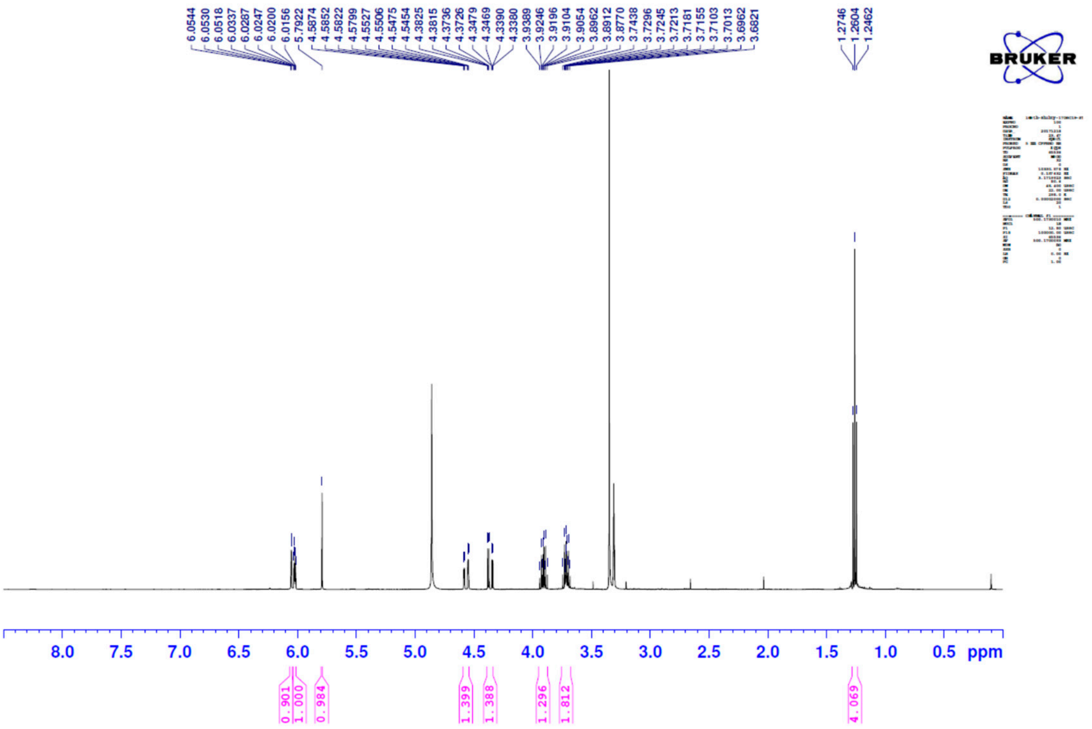

Figure S16. ^{1}H NMR (500 MHz, MeOH-d_4) of **4**.

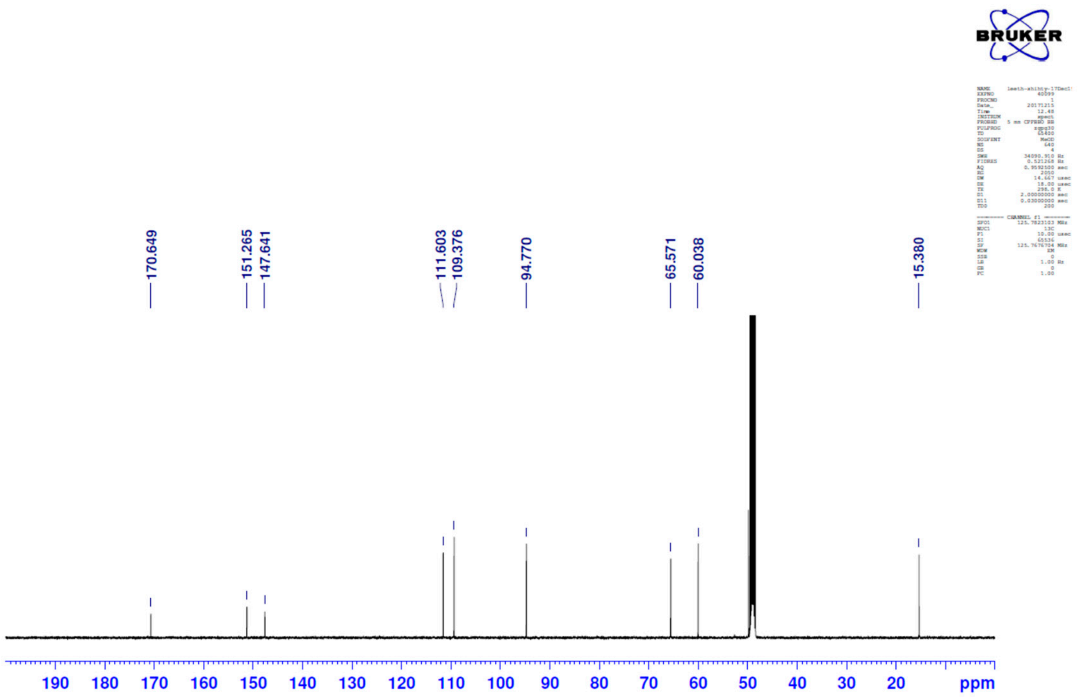

Figure S17. ^{13}C NMR (125 MHz, MeOH-d_4) of **4**.

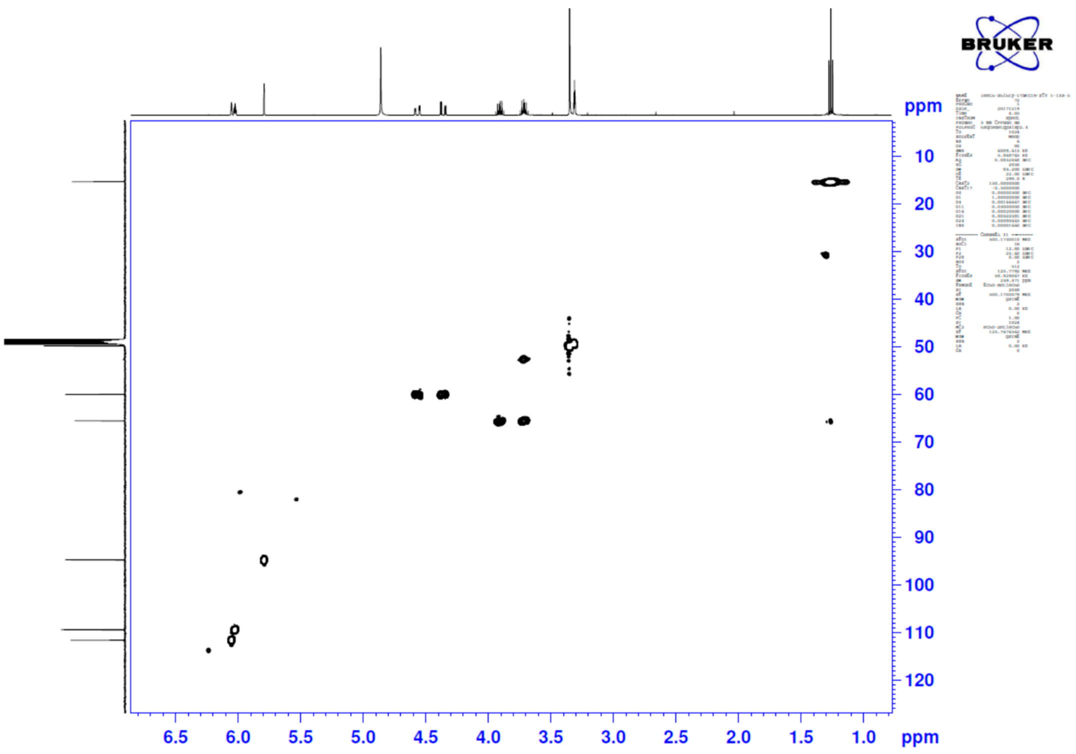

Figure S18. HSQC of **4**.

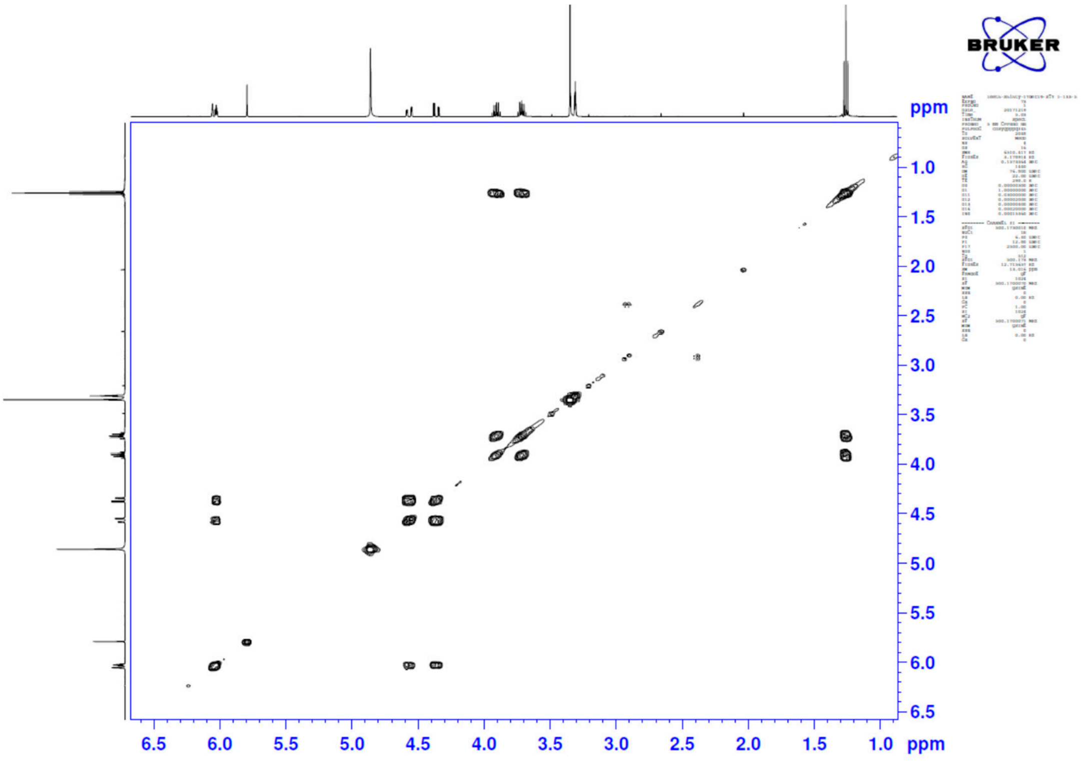

Figure S19. COSY of **4**.

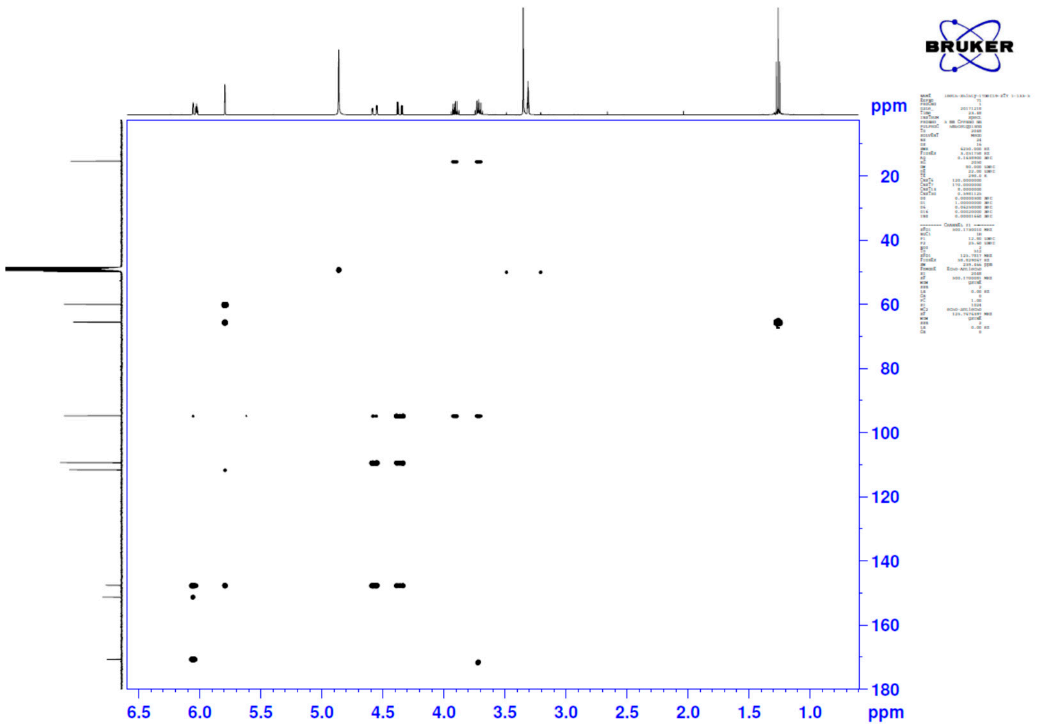

Figure S20. HMBC of **4**.

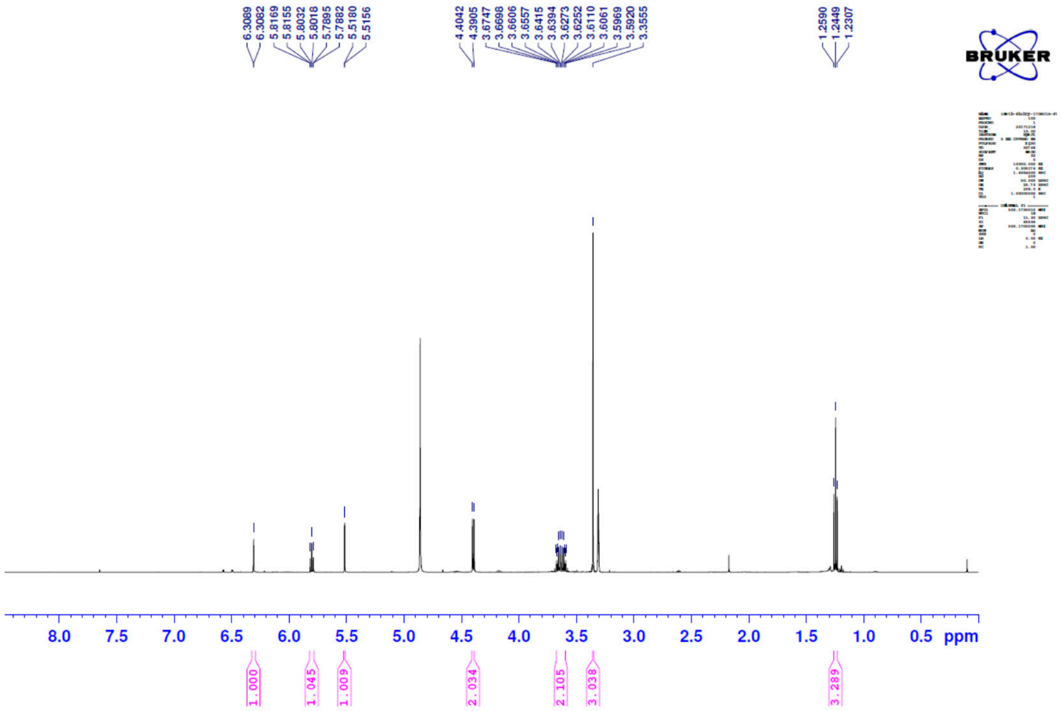

Figure S21. ^{1}H NMR (500 MHz, MeOH-d_4) of **5**.

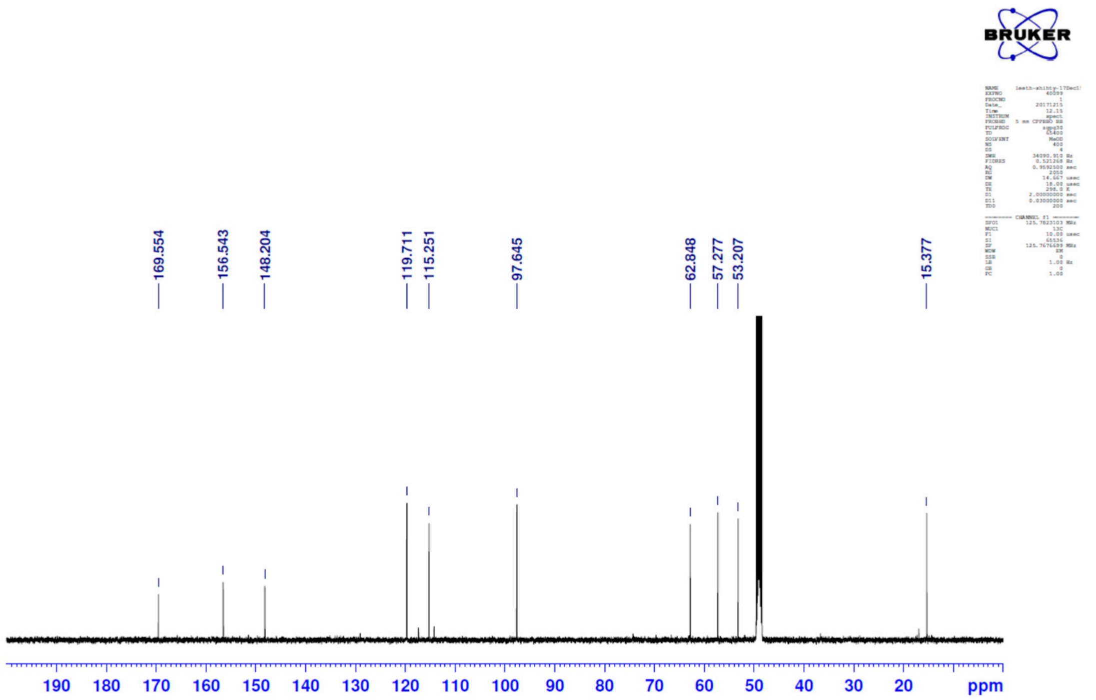

Figure S22. ^{13}C NMR (125 MHz, MeOH-d_4) of **5**.

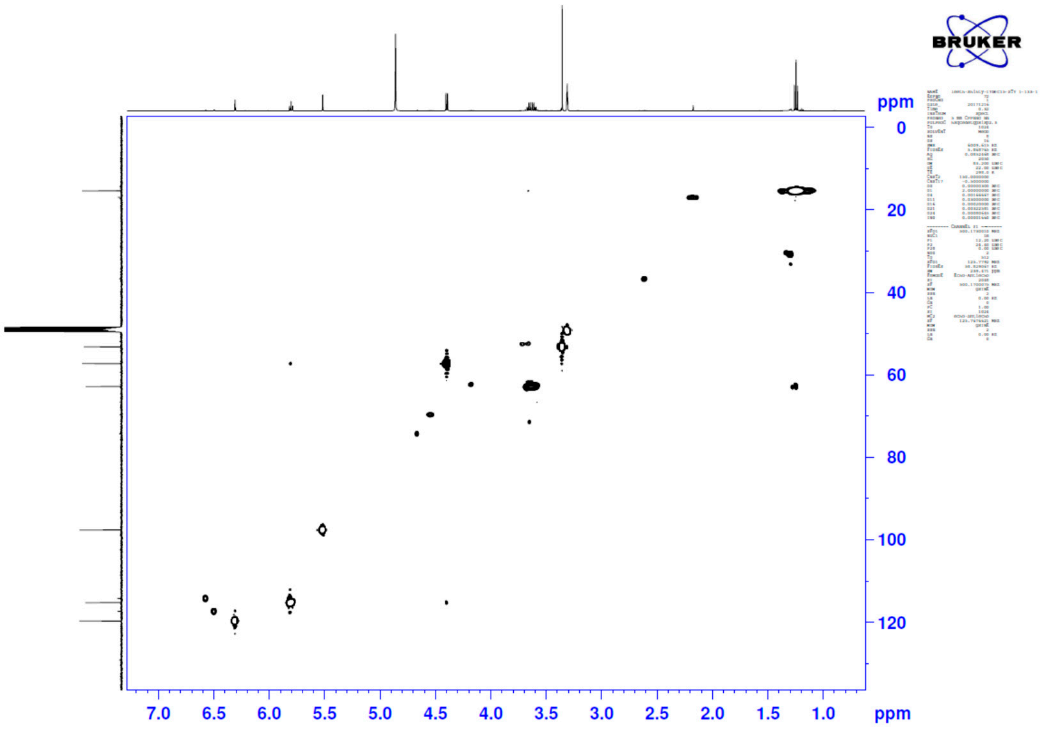

Figure S23. HSQC of **5**.

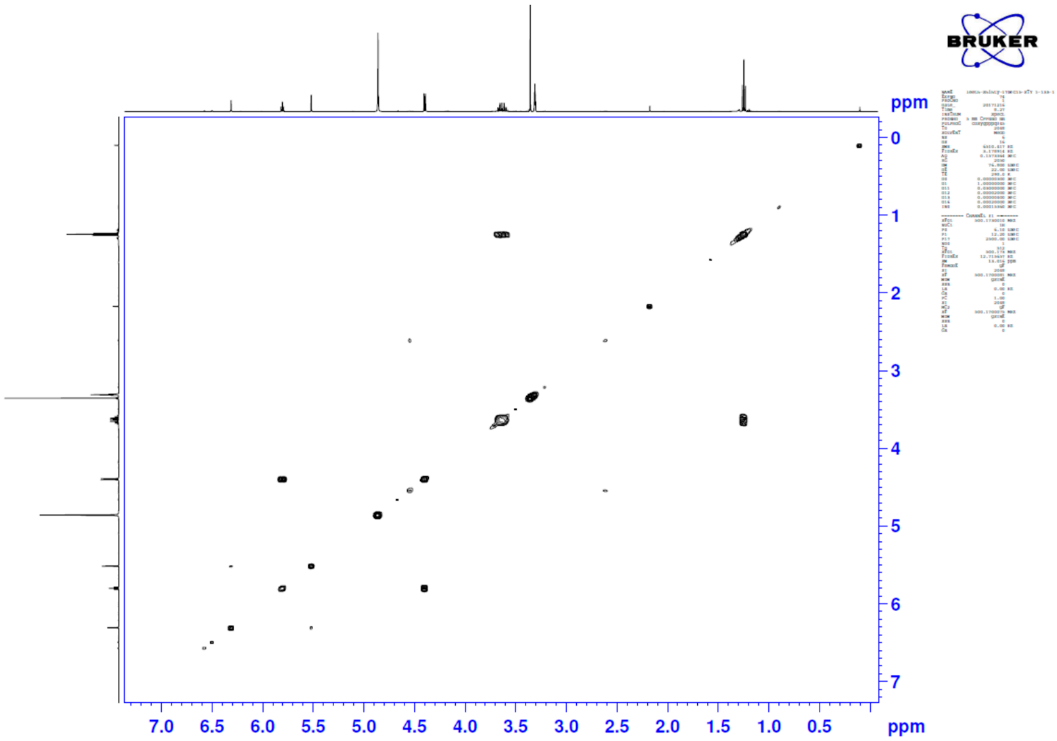

Figure S24. COSY of **5**.

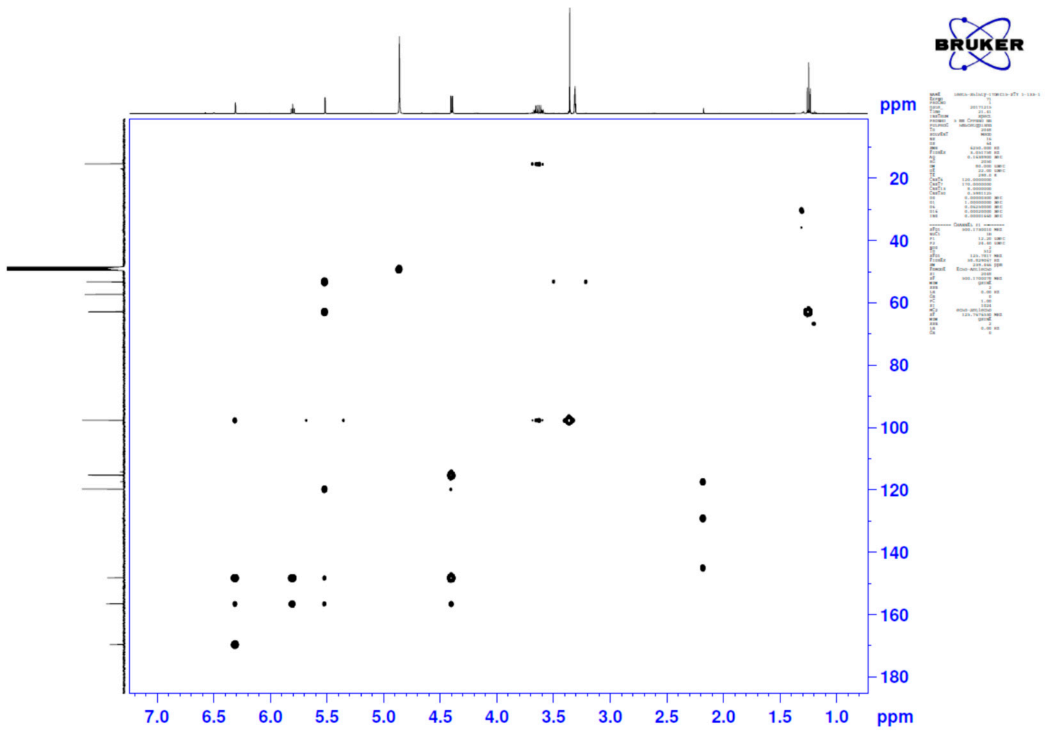

Figure S25. HMBC of **5**.

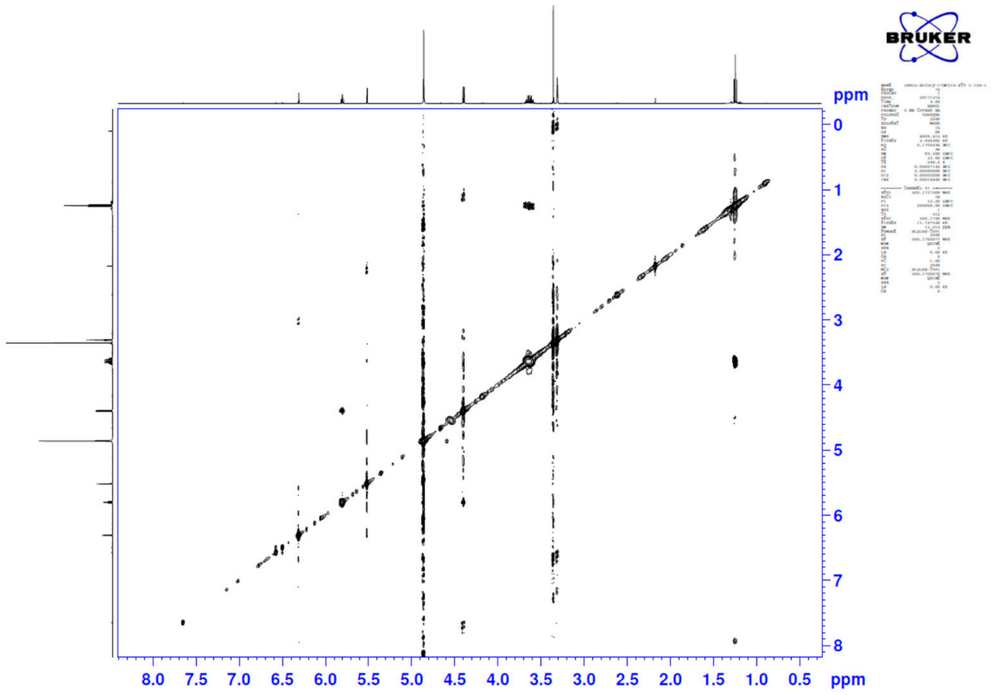

Figure S26. ROESY of **5**.

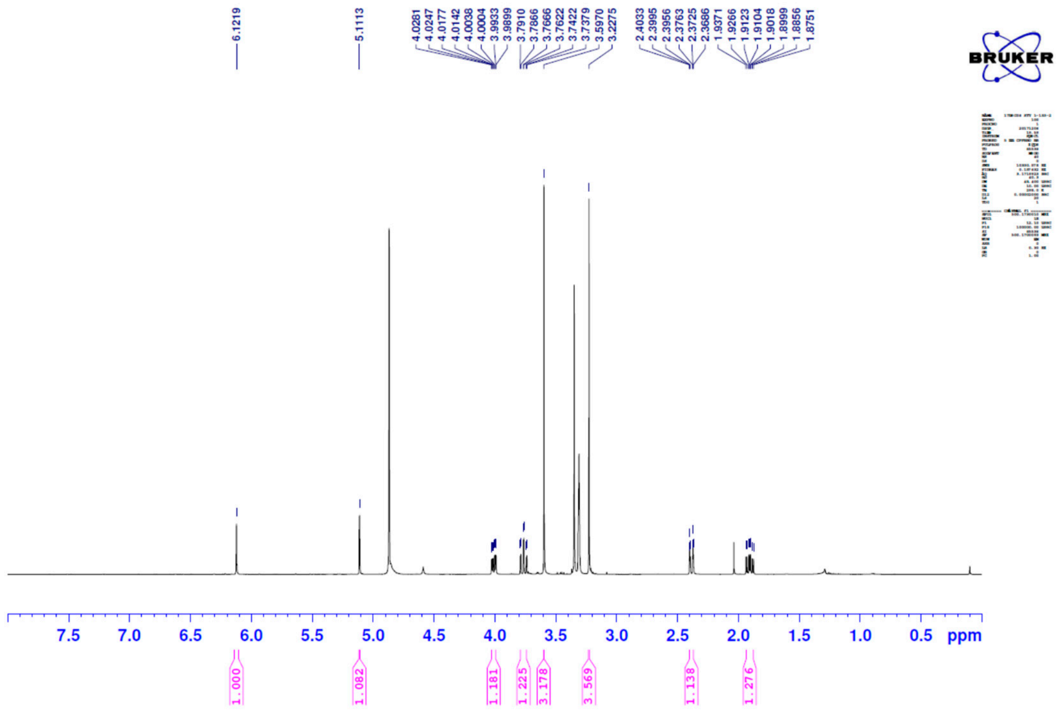

Figure S27. ^{1}H NMR (500 MHz, MeOH-d_4) of **6**.

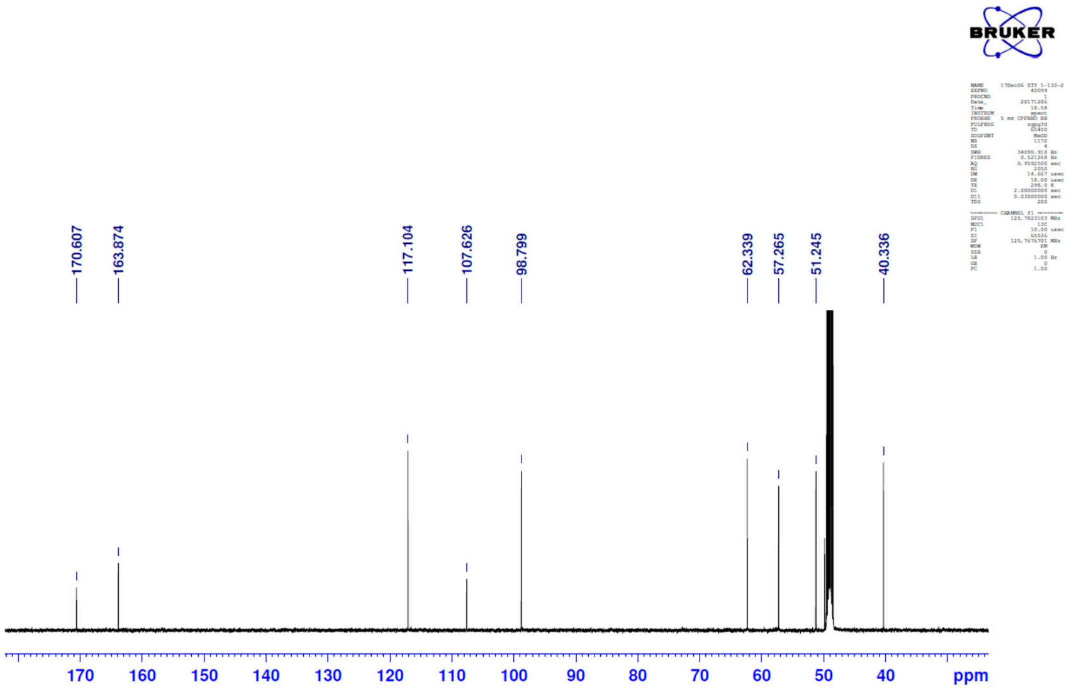

Figure S28. ^{13}C NMR (125 MHz, MeOH-d_4) of **6**.

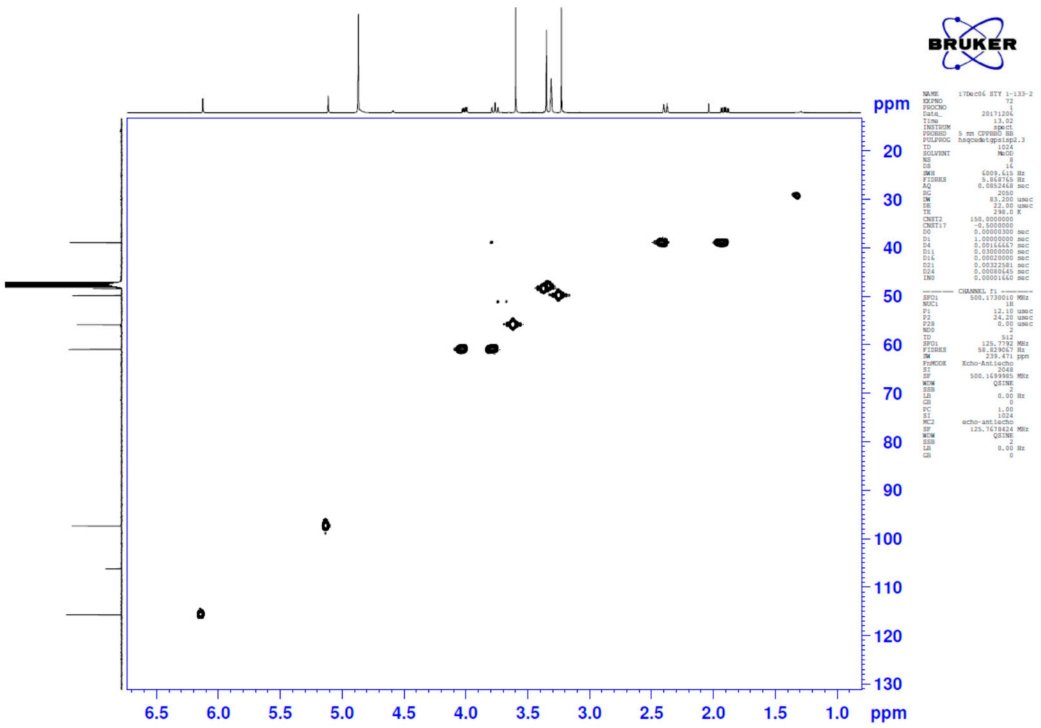

Figure S29. HSQC of **6**.

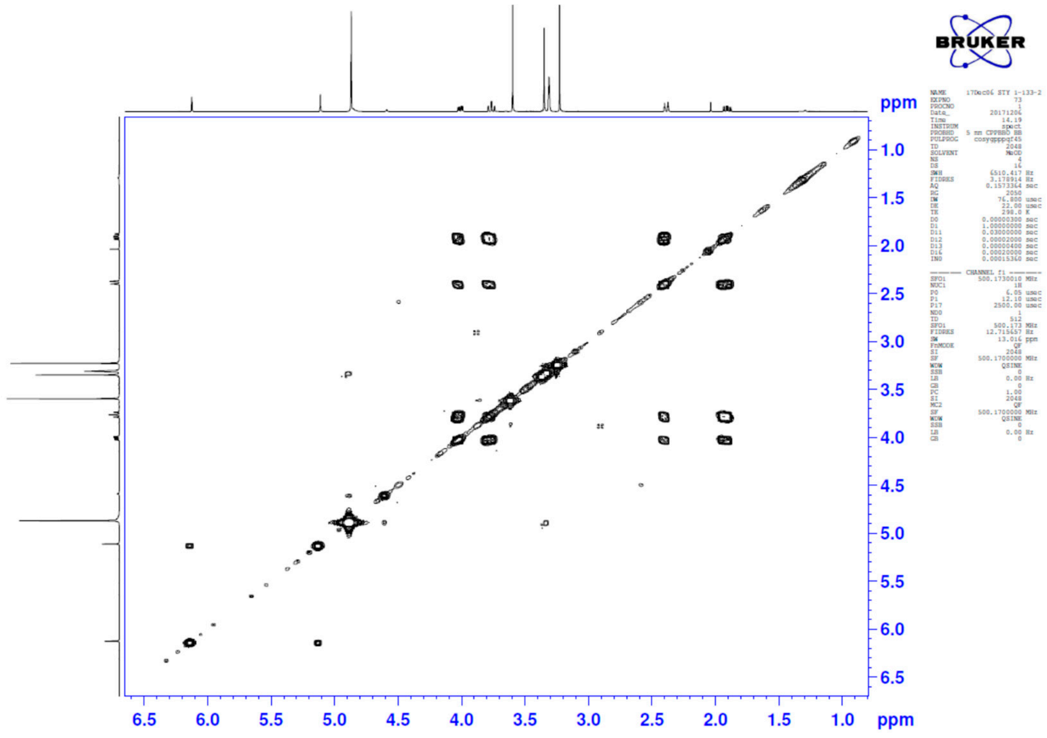

Figure S30. COSY of **6**.

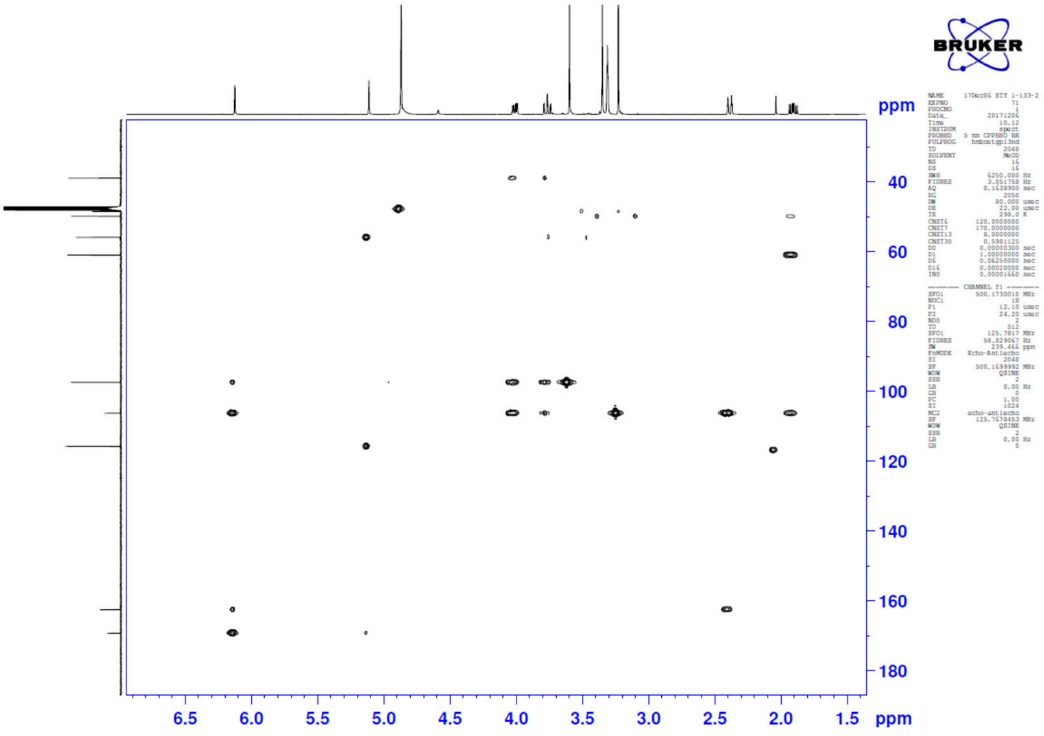

Figure S31. HMBC of **6**.

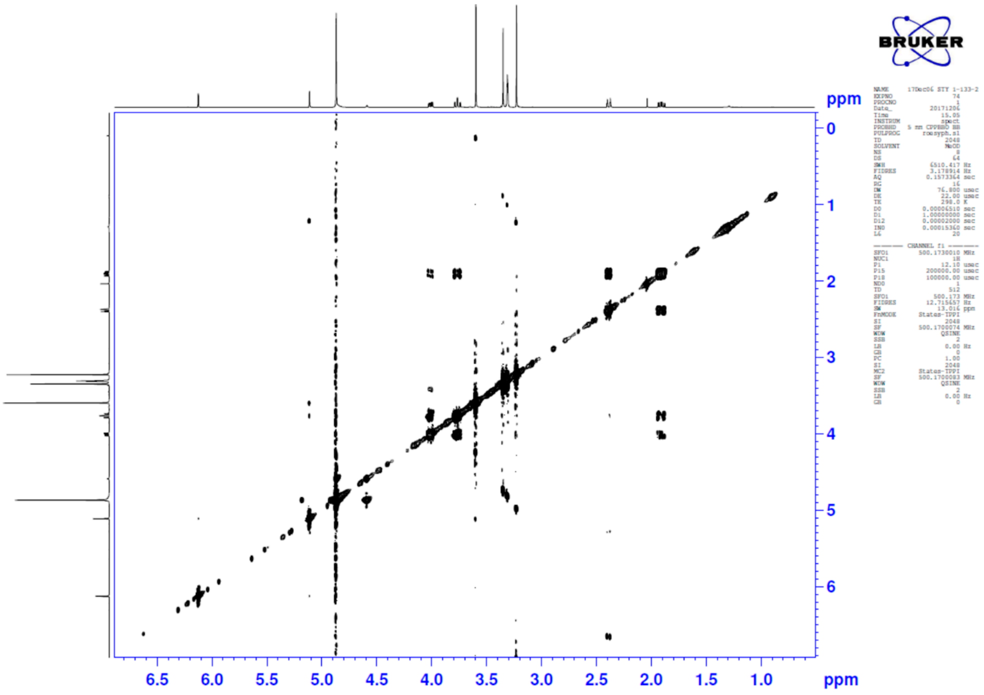

Figure S32. ROESY of **6**.

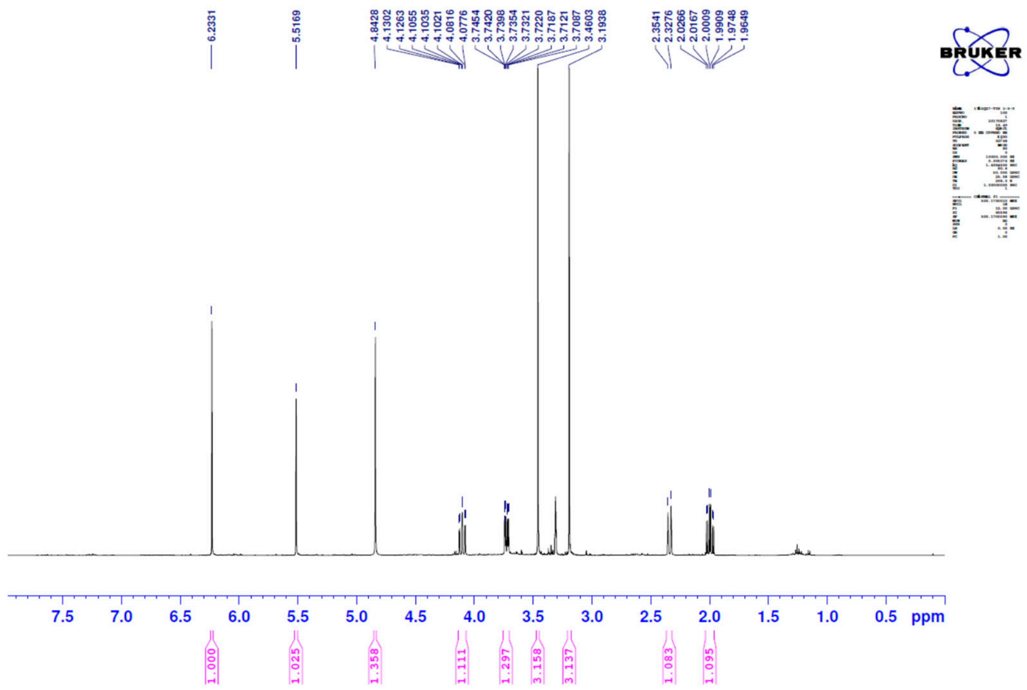

Figure S33. ^{1}H NMR (500 MHz, MeOH-*d*4) of **7**.

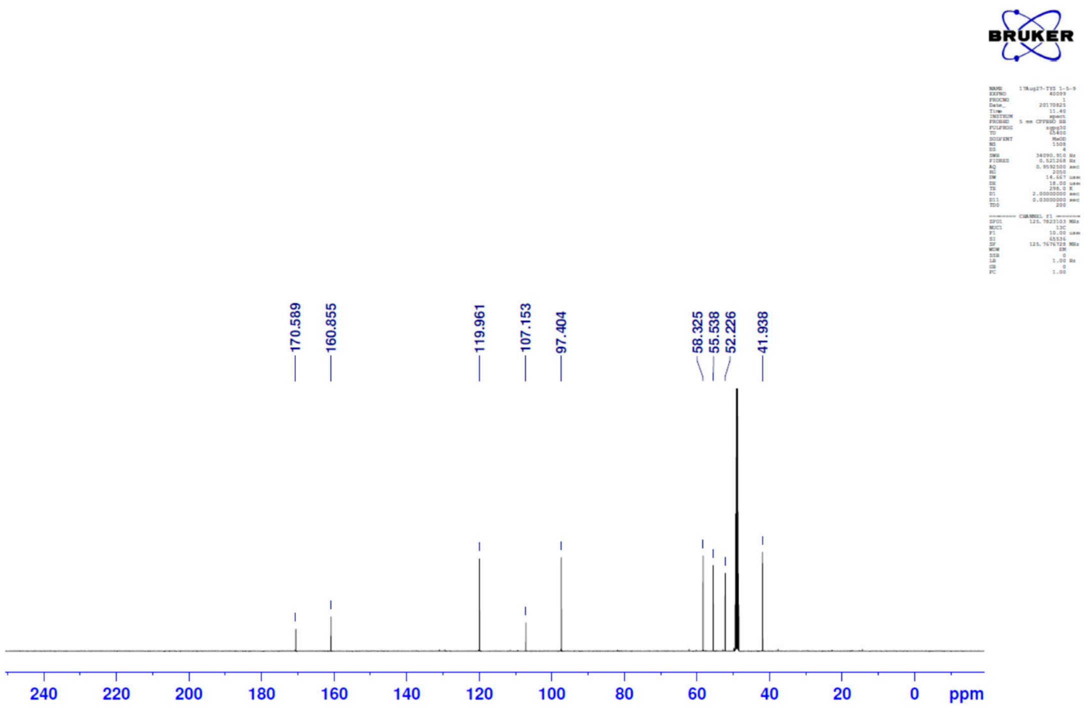

Figure S34. ^{13}C NMR (125 MHz, MeOH-*d*4) of **7**.

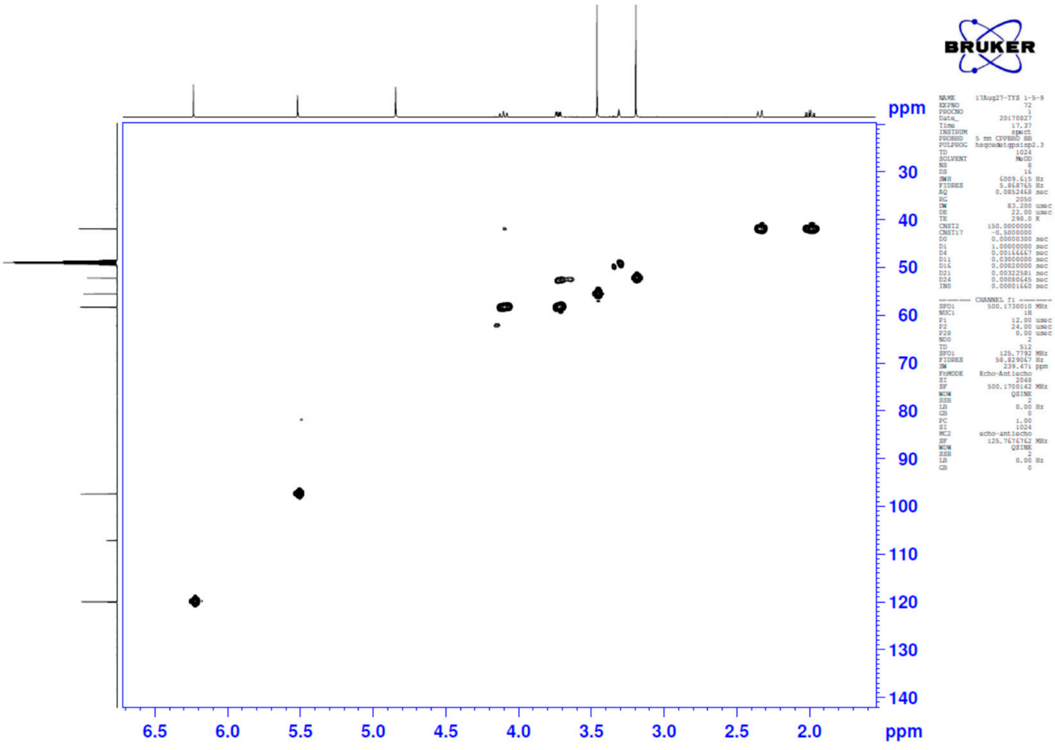

Figure S35. HSQC of **7**.

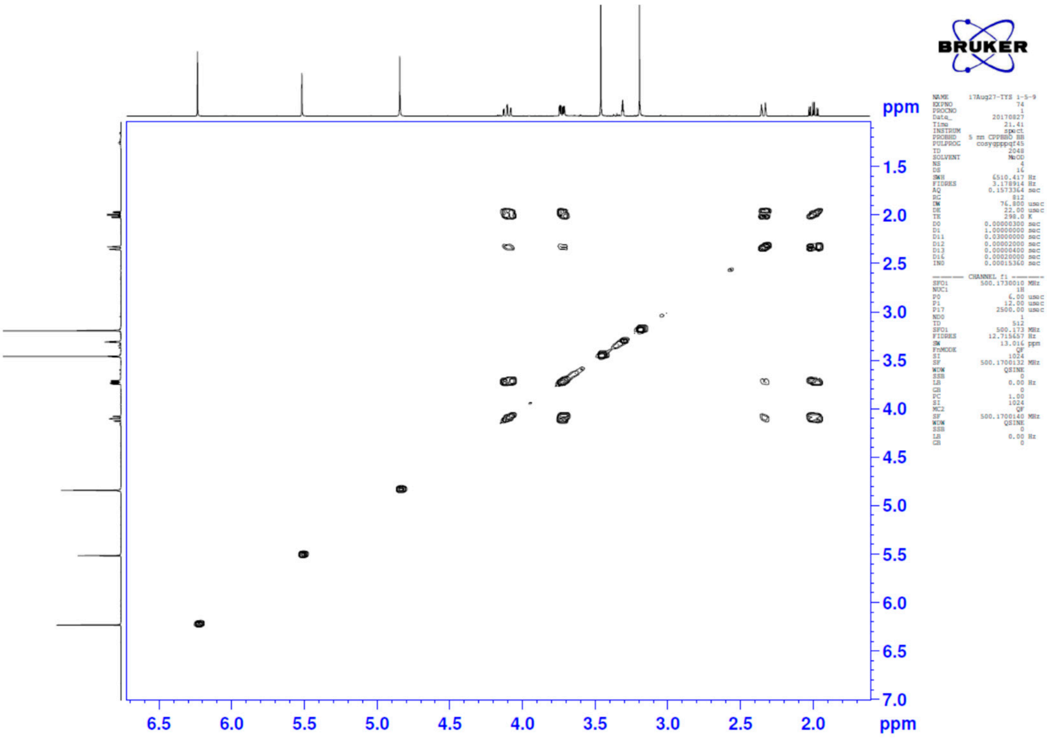

Figure S36. COSY of **7**.

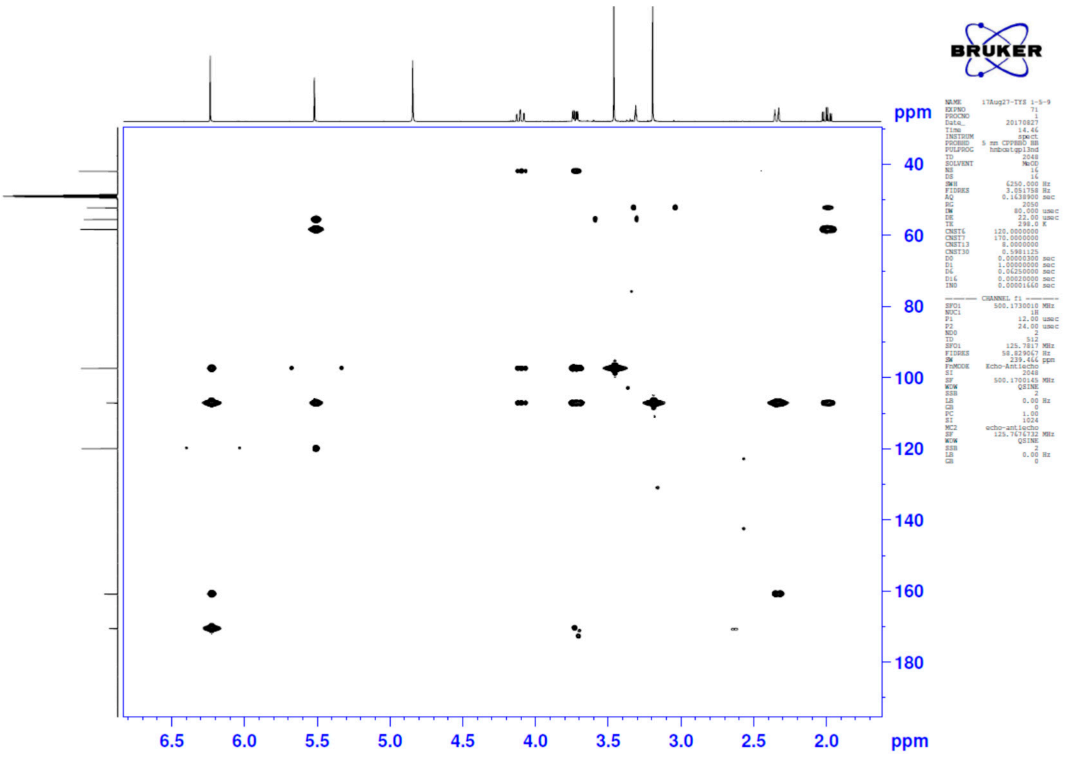

Figure S37. HMBC of **7**.

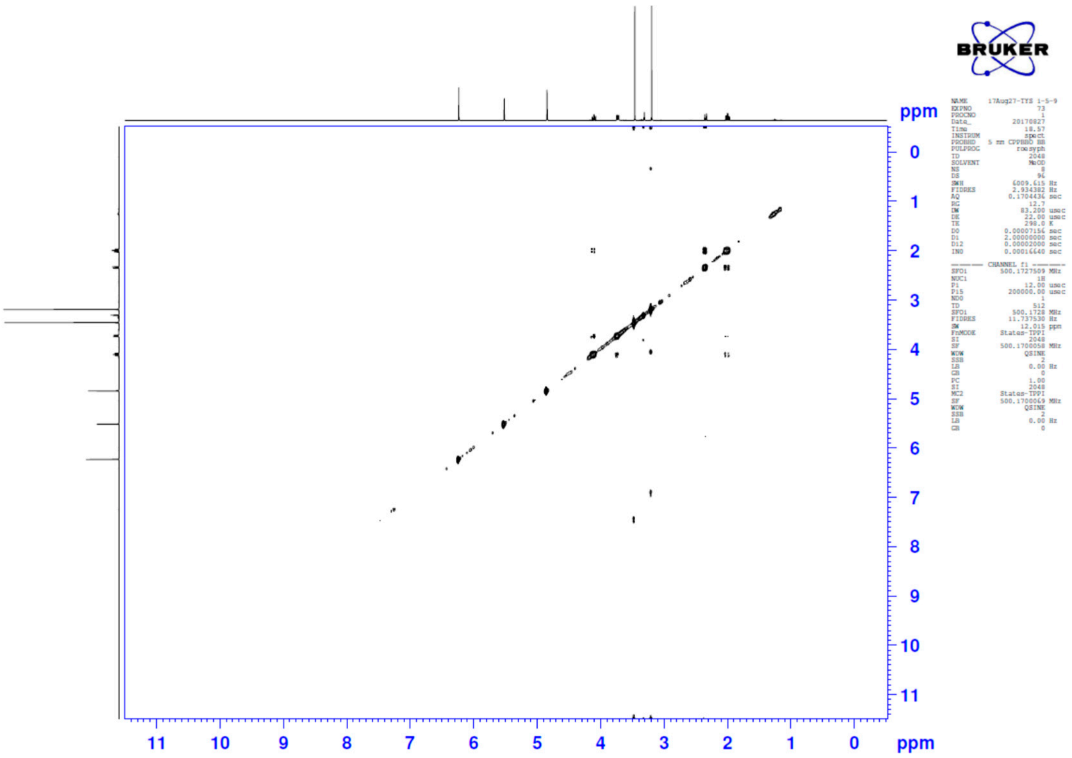

Figure S38. ROESY of **7**.

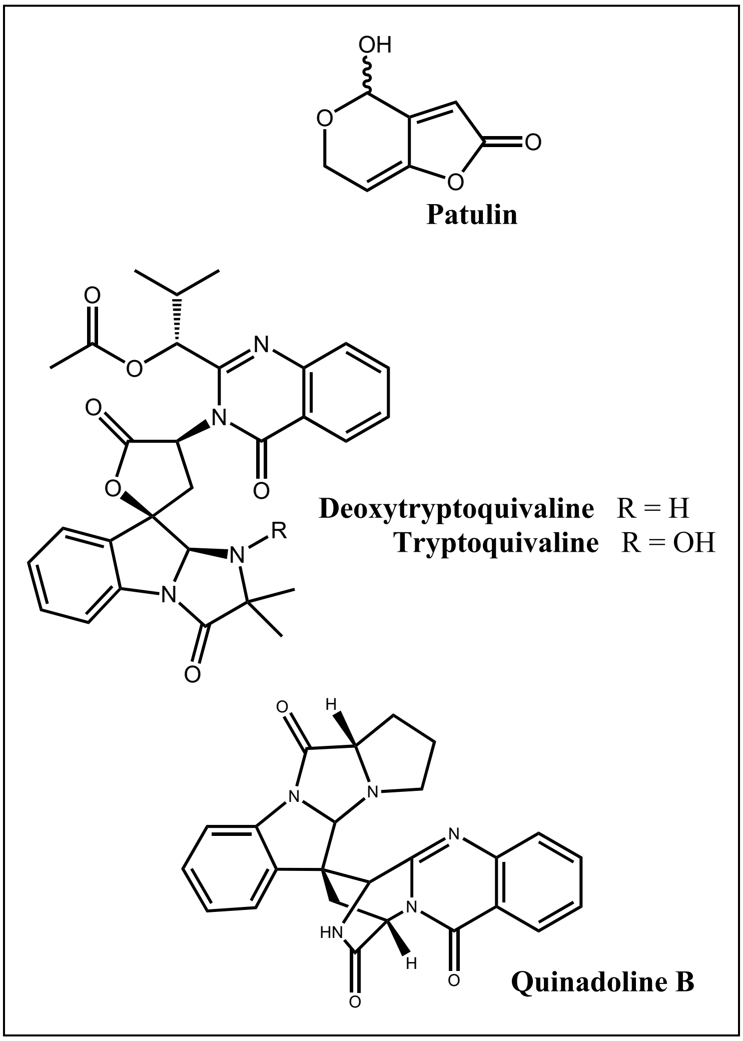

Figure S39. The structures of known compounds isolated in this study.

Table S1. Cytotoxicities of aspergilsmins A-G (**1**-**7**), patulin, deoxytryptoquivaline, tryptoquivaline, and quinadoline B against PC-3 and SK-Hep-1 cells.

Compounds	Cell survival (%)			
	PC-3		SK-Hep-1	
	10 µM	30 µM	10 µM	30 µM
Aspergilsmin A (**1**)	90±2%	96±6%	96±4%	97±6%
Aspergilsmin B (**2**)	93±6%	94±3%	94±2%	96±2%
Aspergilsmin C (**3**)	=0	=0	=0	=0
Aspergilsmin D (**4**)	91±0%	36±1%	94±1%	52±7%
Aspergilsmin E (**5**)	83±2%	18±2%	80±1%	28±2%
Aspergilsmin F (**6**)	100±2%	88±3%	99±3%	97±2%
Aspergilsmin G (**7**)	90±4%	79±2%	90±0%	85±2%
Patulin	=0	=0	=0	=0
deoxytryptoquivaline	90±1%	81±8%	105±2%	104±1%
tryptoquivaline	93±5%	41±8%	103±3%	29±6%
quinadoline B	93±3%	87±2%	101±1%	100±4%
